# Supplementary material for: Deletion of wfs1 Impairs Oligodendrocyte Precursor Cells Dorsal Distribution and Myelination Through the wfs1-hmgcs1 Axis in Zebrafish
Source: Biology (Basel). 2026 Aug 16;15(16):1402. doi: 10.3390/biology15161402 (PMC13510151; doi:10.3390/biology15161402)
Supplement: Supplementary file 1 [file biology-15-01402-s001.zip › File S1-Transcriptome sequencing data and differentially expressed genes (DEGs) in wfs1-- zebrafish at 5 dpf/2.KEGG/wfs1KOvsWT/UP/src/dre04370.html]

dre04370


- dre:793158

- Up regulated genes

793158\_1(1.86160461223548)
- dre:556226

- Up regulated genes

556226\_1(6.36622858283894)

- dre:559020

- Up regulated genes

559020(1.27287729194892)

- dre:565241

- Up regulated genes

565241(1.32977867526604)

- dre:100000252

- Up regulated genes

100000252\_3(1.94434154854557) 100000252(1.09163460881879)

- dre:100006675

- Up regulated genes

100006675(1.63301233543285)

- dre:562552

- Up regulated genes

562552(1.50682290794657)

Close
